# Editorial for the Special Issue “Molecular Biology in Drug Design and Precision Therapy”

**DOI:** 10.3390/cimb48010023

**Published:** 2025-12-25

**Authors:** Cristina Manuela Drăgoi, Ion-Bogdan Dumitrescu, Alina Crenguța Nicolae

**Affiliations:** 1 Department of Biochemistry, Faculty of Pharmacy, Carol Davila University of Medicine and Pharmacy, RO-020956 Bucharest, Romania; cristina.dragoi@umfcd.ro (C.M.D.); alina.nicolae@umfcd.ro (A.C.N.); 2 Department of Physics and Informatics, Faculty of Pharmacy, Carol Davila University of Medicine and Pharmacy, RO-020956 Bucharest, Romania

We are delighted to present this Special Issue of *Current Issues in Molecular Biology*, entitled “Molecular Biology in Drug Design and Precision Therapy.” This collection of research and review articles showcases how cutting-edge molecular biology approaches are driving innovation in drug discovery and enabling more personalized therapeutic strategies. Major advances in recent decades—from high-throughput genomics and proteomics to sophisticated in silico modeling—have paved the way for a paradigm shift in medicine. Central to this evolution is a comprehensive understanding of the molecular underpinnings of disease, coupled with continually improving technological tools, which together allow scientists to design interventions that are more precise and patient-tailored than ever before. In the era of precision medicine, treatments are increasingly being tailored to the unique genetic and molecular profile of each patient, offering the promise of therapies that are more effective and less toxic than the traditional one-size-fits-all approach [1]. Indeed, precision medicine is widely envisioned as the future of healthcare, with the potential to significantly improve patient outcomes by accounting for individual variability in disease pathways and drug responses [2]. At the same time, we must acknowledge that the full realization of this vision is still a work in progress; currently, only a minority of patients benefit from genomics-guided precision therapies, as many diseases lack easily “actionable” molecular targets and drug resistance mechanisms remain a formidable challenge [3]. These realities underscore the importance of ongoing research to expand the arsenal of identifiable biomarkers and druggable targets.

Encouragingly, the rapid expansion of molecular profiling technologies is beginning to address these challenges. Large-scale genomic, transcriptomic, proteomic, and metabolomic analyses now allow clinicians and researchers to characterize diseases with unprecedented granularity. Integrating these multi-layered data is opening the door to truly individualized interventions. For example, beyond genomics alone, considering additional biomarker “layers”—such as epigenetics, imaging, metabolic profiles, microbiome composition, and pharmacogenomics—can significantly enhance predictive power for therapy selection [4]. Advanced computational algorithms (often bolstered by artificial intelligence) can synthesize such complex biomarker information into clinically actionable insights. By bringing together comprehensive molecular datasets, we move closer to the ideal of personalized medicine: a fully tailored treatment strategy based on each patient’s unique biological makeup and circumstances [5]. Notably, this Special Issue itself features studies that leverage a variety of “omics” and systems-biology approaches—from multi-omics analyses identifying novel therapeutic targets to single-cell sequencing elucidating drug mechanisms—reflecting the broader trend of data-driven personalization in medicine.

The field of drug design is also undergoing a transformative evolution, powered by innovative methodologies at the interface of chemistry and molecular biology. This issue arrives on the heels of tremendous recent progress in drug discovery techniques. One notable advance is the rise of “Click Chemistry,” an approach recognized with the 2022 Nobel Prize in Chemistry. Click chemistry enables the rapid and modular synthesis of diverse compound libraries through bio-orthogonal reactions, greatly accelerating the identification of lead compounds. Another breakthrough is Targeted Protein Degradation (TPD) technology, which uses small molecules (such as PROTACs) to harness the cell’s own proteolytic systems to dispose of disease-causing proteins—including many previously deemed “undruggable” by conventional inhibitors. In parallel, DNA-Encoded Libraries (DELs) now allow researchers to screen millions of compounds in a single experiment by linking each candidate molecule to a unique DNA barcode [6]. This massively parallel approach has dramatically increased the efficiency of hit finding and lead optimization in medicinal chemistry. Meanwhile, computer-aided drug design (CADD) and molecular modeling have become indispensable tools in modern drug development, enabling in silico prediction of drug–target interactions and pharmacokinetic properties. With the advent of powerful machine learning techniques, CADD is evolving rapidly—artificial intelligence (AI)-driven models can mine vast chemical and biological datasets to suggest new drug candidates and predict their behavior with improving accuracy [7]. Indeed, looking ahead, AI technologies and interdisciplinary collaboration are poised to tackle the growing complexity of drug discovery, helping to design molecules and optimize therapies in ways that were not previously possible [8]. Collectively, these advanced strategies—from click-chemistry synthesis to AI-guided design—are streamlining the path from bench to bedside, boosting the efficiency and precision of developing new therapeutics.

The contributions in this Special Issue exemplify how such molecular and technological innovations are being applied across the spectrum of biomedical research to yield tangible therapeutic insights. Several papers focus on harnessing the immune system and molecular signaling pathways for next-generation therapies. For instance, one study provides structural and computational insights into tumor immunogenicity, analyzing the peptide motifs and T-cell receptor interactions that distinguish an immunogenic neoantigen from a non-immunogenic one. By elucidating the molecular features that enable effective T-cell recognition, this work offers guidance for cancer vaccine design and T-cell receptor-based therapies in oncology [9]. In another contribution, investigators applied single-cell RNA sequencing to a murine model of systemic lupus erythematosus to understand how a small-molecule drug (the anti-malarial derivative dihydroartemisinin) modulates immune cell heterogeneity. Their findings reveal shifts in splenic T and B cell populations and signaling pathways in treated animals, highlighting potential mechanisms by which this compound ameliorates autoimmunity. Such results not only suggest novel immunomodulatory strategies for lupus, but also demonstrate the power of high-resolution transcriptomic profiling in uncovering drug effects at the cellular level [10]. Similarly, a comprehensive multi-omics analysis in this issue explores the pathogenesis of sepsis and identifies Interleukin-1 Receptor 2 (IL-1R2) as a key immunoregulatory molecule in septic patients. By integrating transcriptomic and pathway data, the authors pinpoint IL-1R2 as a promising precision therapeutic target in sepsis—an important insight for a syndrome historically difficult to treat due to its complex systemic nature. These examples illustrate how deep molecular profiling can yield actionable targets and biomarkers for precision intervention in immunology and inflammation [11].

Other studies in the issue leverage molecular methods to advance drug development from natural products and to overcome drug resistance. One team employed a network pharmacology approach combined with molecular docking and meta-analysis to decipher the therapeutic mechanism of alus, a traditional herbal medicine, in treating acute pancreatitis. They identified multiple bioactive compounds in *Astragalus* and mapped their interactions with key protein targets and pathways (such as PI3K-Akt signaling and inflammatory cascades), providing a scientific basis for the herb’s efficacy and informing its integration into modern therapy [12]. Another intriguing study tackled the problem of chemoresistance in hepatocellular carcinoma, demonstrating that 1,25-dihydroxyvitamin D_3_ (an active form of vitamin D) can reverse tumor resistance to the kinase inhibitor sorafenib. Through a combination of network analysis and experimental validation, the authors showed that vitamin D_3_ modulates the FOXO3/FOXM1 signaling axis and re-sensitizes liver cancer cells to sorafenib, suggesting a novel adjuvant strategy to enhance treatment responses in HCC [13]. Also in the realm of drug resistance, this Special Issue features a structure-based virtual screening study that identified small-molecule antagonists against a viral oncoprotein interaction—specifically, compounds that disrupt the binding of the human papillomavirus E2 and E1 proteins. These in silico findings open a path toward new therapeutics for HPV-related cervical cancer, a disease for which no direct antivirals currently exist [14]. Moreover, addressing the urgent global challenge of antimicrobial resistance, one research article evaluates a novel fluoroquinolone derivative (referred to as compound “7a”) engineered with a difluoroboranyl group. In a murine model of methicillin-resistant *Staphylococcus aureus* (MRSA) pneumonia, this compound showed potent antibacterial activity and significantly reduced lung pathology [15]. Such results underscore the continued importance of medicinal chemistry innovation in developing new antibiotics effective against drug-resistant pathogens.

In addition to original research, this Special Issue contains thought-provoking review and perspective articles that discuss emerging therapies and paradigms. One comprehensive review article on the HIF stabilizer [16] offers an in-depth look at this hypoxia-mimetic drug’s pharmacological effects and clinical applications, as well as the methods for detecting its misuse in sports. Roxadustat’s story—from treating anemia by inducing erythropoietin to concerns about doping—exemplifies the multifaceted considerations in modern drug development, spanning therapeutic benefit, safety, and regulatory oversight. Another timely review focuses on cefiderocol, a novel siderophore cephalosporin antibiotic sometimes nicknamed a “Trojan horse” for its strategy to penetrate Gram-negative bacteria by hijacking iron transport pathways. The review summarizes cefiderocol’s potent activity against multidrug-resistant bacteria, while also alerting readers to the emerging mechanisms of resistance that could limit its efficacy [17]. Such discussions are crucial in guiding the optimal use of new anti-infectives and in spurring the search for next-generation compounds. Finally, a forward-looking perspective article in this issue proposes the “EC5” multi-drug regimen—a combination of five repurposed, off-patent medications (alendronate, celecoxib, itraconazole, ramelteon, and simvastatin)—as an adjunct to standard therapy in refractory endometrial cancer. This imaginative approach is grounded in molecular rationale: each drug in the EC5 cocktail is hypothesized to target a specific pathway involved in cancer progression (from inflammation to metabolism and immunity) [18]. While investigational, such a strategy embodies the creative repurposing of existing drugs based on mechanistic insights, a concept that is gaining traction to accelerate therapy development by leveraging known pharmacologies.

In summary, the papers assembled in this Special Issue span a remarkable range of disease areas and methodologies, yet they share a unifying theme—the power of molecular biology to propel drug design and precision therapy. From cancer and autoimmune disorders to infectious and metabolic diseases, deep molecular insight is the common denominator for innovation across all domains of medicine. The studies herein highlight how integrating diverse scientific disciplines—biochemistry, genomics, pharmacology, bioinformatics, and beyond—can lead to concrete advances: be it a new drug candidate, a novel therapeutic target, or a refined treatment strategy tailored to patient subgroups. Equally important, they remind us that translating molecular findings into clinical impact often requires a multidisciplinary effort and a consideration of real-world complexity (such as variability in patients or pathogen resistance). As we look to the future, we are optimistic that ongoing developments in technology (like AI and next-gen sequencing), in combination with global collaborative research, will continue to accelerate the discovery of targeted therapies and the implementation of precision medicine. The frontier is broad—spanning improved vaccines and immunotherapies, gene and cell therapies, smart drug-delivery systems, and personalized treatment algorithms—but each step forward begins with understanding the fundamental biology of disease.

We sincerely hope that the readers find this Special Issue informative and inspiring. The Guest Editors would like to thank all the authors for contributing their cutting-edge work to this collection and the peer reviewers for their insightful feedback that helped improve each manuscript. By sharing these advances, we aim to stimulate further research and collaboration in the community, ultimately driving the field of molecular medicine toward more effective and individualized patient care.

## References

[1] Nygren P. (2025). Precision cancer medicine 2025: Some concerns. Acta Oncol..

[2] Drăgoi C.M., Diaconu C.C., Nicolae A.C., Dumitrescu I.B. (2024). Redox Homeostasis and Molecular Biomarkers in Precision Therapy for Cardiovascular Diseases. Antioxidants.

[3] Nicolae A.C., Arsene A.L., Vuță V., Popa D.E., Sîrbu C.A., Dragomiroiu G.T.A.B., Dumitrescu I.-B., Velescu B., Gofiță E., Dragoi C.M. (2016). In Vitro P-GP Expression after Administration of CNS Active Drugs. Farmacia.

[4] Diaconu C.C., Cozma M.A., Dobrică E.C., Gheorghe G., Jichitu A., Ionescu V.A., Nicolae A.C., Drăgoi C.M., Găman M.-A. (2021). Polypharmacy in the Management of Arterial Hypertension—Friend or Foe?. Medicina.

[5] Cummings J.L., Teunissen C.E., Fiske B.K., Le Ber I., Wildsmith K.R., Schöll M., Dunn B., Scheltens P. (2025). Biomarker-guided decision making in clinical drug development for neurodegenerative disorders. Nat. Rev. Drug Discov..

[6] An Q., Huang L., Wang C., Wang D., Tu Y. (2025). New strategies to enhance the efficiency and precision of drug discovery. Front. Pharmacol..

[7] Yu W., MacKerell A.D. (2017). Computer-Aided Drug Design Methods.

[8] Berg E.L. (2021). The future of phenotypic drug discovery. Cell Chem. Biol..

[9] Doytchinova I., Sotirov S., Dimitrov I. (2025). Molecular Insights into Tumor Immunogenicity. Curr. Issues Mol. Biol..

[10] Qin H., Zhu X., Liu X., Wang Y., Liang J., Wu H., Wu J. (2025). Dihydroartemisinin Alleviates the Symptoms of a Mouse Model of Systemic Lupus Erythematosus Through Regulating Splenic T/B-Cell Heterogeneity. Curr. Issues Mol. Biol..

[11] Dave K., Munteanu C.R. (2025). IL-1R2 as a Precision Therapeutic Target in Sepsis: Molecular Insights into Immune Regulation. Curr. Issues Mol. Biol..

[12] Cao X., Duan S., Li A., He Z. (2025). Astragalus in Acute Pancreatitis: Insights from Network Pharmacology, Molecular Docking, and Meta-Analysis Validation. Curr. Issues Mol. Biol..

[13] Long Z., Wu X., Luo T., Chen X., Huang J., Zhang S. (2025). Exploring the Molecular Mechanism of 1,25(OH)2D3 Reversal of Sorafenib Resistance in Hepatocellular Carcinoma Based on Network Pharmacology and Experimental Validation. Curr. Issues Mol. Biol..

[14] Gandara-Mireles J.A., Loera Castañeda V., Grijalva Ávila J.C., Villanueva Fierro I., Muñoz C.M., Payan Gándara H., Romero L.P., Reyes H.A. (2025). In Silico Identification of Potential Antagonists Targeting the HPV16 E2-E1 Interaction: A Step Toward Novel Therapeutics for Cervical Cancer. Curr. Issues Mol. Biol..

[15] Veyna-Hurtado L.A., Hernández-López H., Reyes-Escobedo Fdel R., de Loera D., García-Cruz S., Troncoso-Vázquez L., Galván-Valencia M., Castañeda-Delgado J.E., Cervantes-Villagrana A.R. (2025). The Derivative Difluoroboranyl-Fluoroquinolone “7a” Generates Effective Inhibition Against the *S. aureus* Strain in a Murine Model of Acute Pneumonia. Curr. Issues Mol. Biol..

[16] Creangă E.C., Ott C., Nicolae A.C., Drăgoi C.M., Stan R. (2025). Roxadustat as a Hypoxia-Mimetic Agent: Erythropoietic Mechanisms, Bioanalytical Detection, and Regulatory Considerations in Sports Medicine. Curr. Issues Mol. Biol..

[17] Bianco G., Boattini M., Cricca M., Diella L., Gatti M., Rossi L., Bartoletti M., Sambri V., Signoretto C., Fonnesu R. (2024). Updates on the Activity, Efficacy and Emerging Mechanisms of Resistance to Cefiderocol. Curr. Issues Mol. Biol..

[18] Kast R.E. (2025). Potential Benefits of Adding Alendronate, Celecoxib, Itraconazole, Ramelteon, and Simvastatin to Endometrial Cancer Treatment: The EC5 Regimen. Curr. Issues Mol. Biol..

